# Multidimensional well-being and income inequality in Central and Eastern Europe: A comparative analysis of CEE North and CEE Continental countries

**DOI:** 10.1371/journal.pone.0316325

**Published:** 2025-01-14

**Authors:** Andrzej Geise, Małgorzata Szczepaniak

**Affiliations:** 1 Department of Econometrics and Statistics, Faculty of Economic Sciences and Management, Nicolaus Copernicus University in Torun, Torun, Poland; 2 Department of Economics, Faculty of Economic Sciences and Management, Nicolaus Copernicus University in Torun, Torun, Poland; University of Maribor Faculty of Arts: Univerza V Mariboru Filozofska Fakulta, SLOVENIA

## Abstract

Central Eastern European countries (CEEc) are characterized both by huge diversity in income inequality and, on average, by lower levels of well-being than in the other European Union (EU) countries. Given that income inequality may affect well-being negatively, the present study aims to explore the links between income inequalities and different dimensions of well-being in the eight CEEc, i.e. Poland, Czech Republic, Slovakia, Slovenia, Hungary, Latvia, Lithuania, and Estonia. The analysis is conducted in the two groups of CEEc regarding low and high inequality in income distribution, namely CEE Continental group and CEE North group (corresponding to post-socialist corporatist and post-socialist liberal, respectively). The multidimensional concept of well-being is applied, enabling deep exploration of its links with income inequalities in the following dimensions: subjective well-being (happiness) and objective well-being (material, health, educational, and environmental dimensions). We estimate the vector autoregression (VAR) models based on annual data disaggregated into quarterly data covering 2004 to 2020. The empirical results of Granger causality testing, which was used to investigate the links between income inequality and multidimensional well-being, indicated that not only are there differences between the groups in the studied patterns of interconnectedness, but also the groups of CEE North and CEE Continental countries are not homogeneous in those links.

## Introduction

Economic integration in the European Union (EU) has slowed, raising concerns over income inequalities between and within countries over the past decade. If the EU was considered a single country, income inequality across all its EU citizens would have declined over time, but the levels of income inequality between the richest and the poorest countries continue to be vast [1]. Moreover, Central and Eastern European countries (CEEc), as members of the EU since 2004, constitute an interesting phenomenon when income inequalities are considered. Those post-socialist states have a similar historical background and stronger preferences for equality [2] than Western European countries. However, despite a common, increasing trend in income inequalities from the beginning of socio-economic transformation since the demise of communist rule in the late 1980s, the vast diversity in income inequalities across the CEEc has been observed after about three decades of their economic transformation, with the Gini index ranging from 21.8 in Slovakia to 35.7 in Latvia 2021 [3]. Therefore, following the World Bank Report on the European Union [1], Eurofound Report [4], and [5] classifications of CEEc concerning welfare states regime, in the present study, the CEEc are divided into two groups distinguished based on differences in the level of income inequalities. The first group, CEE North, constitutes the CEEc with relatively high (above-average) income inequality levels, i.e., Latvia, Lithuania, and Estonia. The second group, namely CEE Continental, consists of CEEc with relatively low (lower than average) levels of income inequalities, i.e., Slovenia, Slovakia, Hungary, Czech Republic, and Poland. A similar classification was applied by Whelan and Maitre [5] in the analysis of life satisfaction inequalities between European countries, which was based on the welfare state typology developed by Esping-Andersen [6]. Their study grouped CEEc into post-socialist corporatist and post-socialist liberal clusters (CEE Continental and CEE North, respectively; [5]. Combining criteria of the strictness of employment protection legislation with those reflected in the standard Esping-Andersen categorization and expanding the coverage to include the newer European Member States that joined in 2004, Eurofound in their classification [4] distinguished post-socialist corporatist and post-socialist liberal among six welfare state regimes. The post-socialist corporatist regime comprises the central European countries (Czech Republic, Hungary, Poland, Slovenia, and Slovakia) with mostly transfer-oriented labor market measures and a moderate degree of employment protection, which in our study corresponds to CEE Continental with relatively low-income inequalities level. The post-socialist liberal cluster comprises the Baltic states (Estonia, Latvia, and Lithuania), which are characterized by a more flexible labor market [4, 5] and, in our study, correspond to CEE North, characterized by relatively high-income inequality levels.

On the other hand, apart from high diversity in income inequality, CEEc have been characterized by a relatively lower level of well-being in comparison to other European, i.e. liberal and conservative-corporatist Western European countries [7], supposedly because of social problems connected with the systemic transformation that limited the advances in well-being [8]. Moreover, considering well-being as a multidimensional phenomenon [9–11], the issue becomes even more complex when both subjective and objective measures are analyzed. For instance, the European Commission’s ’Going Beyond GDP’ initiative well-being should be evaluated by considering well-being indicators that are beyond material dimension but should also consider such aspects as health, education, and environmental factors among other dimensions [12]. In the context of sustainable development, because of a growing concern that income inequalities can negatively affect well-being [13], our research addresses the following global problems and Sustainable Development Goals (SDGs), i.e. income inequalities (SDG No.10), well-being in the different dimensions, namely health (SDG No.3), education quality (SDG No.4), and natural environment (SDG No.13).

Given that income inequality may have harmful consequences for well-being [14] and that CEEc is characterized both by huge diversity in income inequality and, on average, by lower than in UE level of well-being, the present study aims to explore the links between income inequalities and different dimensions of well-being in the selected eight CEEc in two groups. Following the classifications of CEE North and CEE Continental and the corresponding differences in the income inequality levels in those groups, the current study aims to disentangle the relationships between income inequalities and different dimensions of well-being and compare the results in two groups characterized by low and high-income inequalities. To conduct an in-depth analysis, we do not average the data in the groups but conduct the analysis for each country in the delimited group separately. Such an approach makes it possible to check if the countries in the groups are homogenous in terms of similar patterns of relationships between income inequalities and dimensions of well-being. The following research questions pertaining to the links between income inequalities and different dimensions of well-being in the delimited CEEc groups are studied:

RQ1: Are there differences in the identified causality patterns between economies of CEE North and CEE Continental countries groups?
RQ2: Are there similarities in the direction of the response of income inequalities to changes in well-being dimensions between the economies of CEE North and CEE Continental groups?
RQ3: Which dimensions of well-being bear the burden in explaining variation in income inequality in both groups of CEE countries?

Overall, the contributions of this article are twofold. Firstly, the present study incorporates the multidimensional well-being concept to study the links with income inequality measures for CEE countries, which goes beyond only one-dimension comparisons (i.e., material or subjective well-being) and is therefore in line with the sustainable development and beyond GDP concept. Moreover, the assumption of the multi-faceted construct of well-being allowed the in-depth study of the different relationships between income inequalities and the following dimensions of well-being: subjective (happiness) and objective (material, health, educational, and environmental). We use the time series approach (the vector autoregressive model VAR) incorporating causality analysis, the impulse response function and the forecast error variance decomposition for each country separately to evaluate the impact of disaggregated well-being dimensions on income inequalities. At the same time, most studies focus on a group of countries using panel models whose underlying fundamental homogeneity assumptions have been called into question. Such a novel approach not only enabled us to avoid uncovering the important relations that could have been missed when an overall measure of well-being was studied but also allowed us to identify important associations between dimensions of well-being and their links with income inequalities in a more detailed analysis. Moreover, the applied methodology allowed us to deepen the previous analysis on the links between income inequalities and multidimensional well-being in the short and long term, with general conclusions for the CEEc [15]. Based on the research conducted with the new methodology, policy recommendations were formulated and adjusted for particular relationships and CEEc groups of highly different income inequality levels. The present study filled, therefore, the identified gap. Secondly, because of the identified diversity of income inequalities in the eight CEEc (Poland, Czech Republic, Slovakia, Hungary, Latvia, Lithuania, and Estonia) and to avoid generalizing the results, we decided to study the links separately for each country and to compare in the two groups of CEE countries, divided in terms of income inequality level, namely CEE North and CEE Continental.

The remainder of the present article is organized as follows. Section II discusses the literature concerning the links between income inequalities and selected dimensions of well-being. The data and inequality trends are described in Section III. The methods and the results of the empirical analysis are presented in Section IV. The last Section offers the discussion and concluding remarks.

## Literature review

Even though a vast part of the studies indicate negative relationships between income inequalities and well-being [14, 16–18], the results concerning the directions and strength of those links are ambiguous in the literature. Possible explanations are that there is no consensus on what well-being means [19] and complexity of the dimensions it covers [20]. Well-being is considered an evaluation of the quality of life, reflecting not only living conditions but also how people respond to their lives in the life domains [21]. It is often underlined that apart from objective dimensions of well-being (income, job, environment, health, institutions), the subjective well-being, i.e. life satisfaction, happiness, plays important role [22–24]. One of the philosophical approaches to subjective well-being is prudential happiness, which is achieved when a person achieves a high state of well-being, both mentally and physically [24], and is often used interchangeably with terms subjective well-being [25].

However, the multidimensional approach to well-being is relatively rarely applied in terms of the relationships with income inequalities. Moreover, even though some authors used the broad concept of well-being, [15, 26, 27] the established relationships are highly sensitive to the selection of countries [16] and the set of dimensions of well-being studied [28]. The study [27] analyzed eleven well-being indicators from the OECD Better Life Index in the European regions in 2000 and 2014; the well-being interactions with income inequalities differed, accounting for their being seen as complements or substitutes. The negative relations between income inequalities and well-being in the personal and community well-being categories were shown [27]. The study [26] uses the entropy method to divide the items of the Better Life Index into four categories, i.e. economic, environmental, social, and well-being. The last one is calculated based on life expectancy, self-reported health, life satisfaction, time devoted to leisure, and the share of employees working very long hours is evaluated as relatively low efficiency in CEEc in comparison to other OECD countries [26]. Szczepaniak and Geise [15] identified long-run and short-run associations between selected dimensions of well-being and income inequalities for the group of eight CEEc in 2004–2018. The results of their study revealed that in the long run, all the dimensions of well-being negatively affected unequal distribution, whereas the positive impact of income inequalities on material dimension and education was revealed. Also, the short-run direct influence of income inequalities on health and happiness was identified [15]. However, the results obtained based on the panel ARDL model are average for the group of CEE countries, and the authors could not identify the diversity in the income inequalities and well-being dimensions between studied countries.

Differing results found in various studies underscore that income inequality does not affect all dimensions of well-being equally. Moreover, those associations may differ regarding income inequality levels in particular economies. According to [29], people living in more unequal countries generally report higher well-being than those from more equal countries. This association, however, does not apply to all societies universally. The positive effect of a nation’s income inequality is weaker when individuals underscore egalitarian norms to a more considerable extent [29].

Because CEE countries have stronger preferences for equality because of systemic similarities before the transformation [2], an in-depth analysis of the income inequalities and well-being associations is needed. Further differences in the level of income inequalities in the group of post-socialist countries make the issue more important. However, to the authors’ knowledge, no such study focuses on this issue regarding differences in income inequalities in CEE countries.

Most studies focus on the links between only one selected dimension of well-being and income inequalities. Most often, subjective well-being, namely life satisfaction, is considered concerning equality in income distribution [30, 31]. Respectless relatively broad literature on the relationships between income inequalities and subjective well-being, no consensus exists. Ngamaba et al., [32] based on a meta-analysis of twenty-four studies, revealed no association. However, their subgroup analysis followed Schneider’s results [33] and Ferrer-i-Carbonell and Ramos’ [34] negative associations in European countries. Possible reasons for this ambiguity are different concepts of defining and measuring subjective dimensions of well-being, i.e. life satisfaction, happiness, and the country‘s economic development. The results of Ngamaba et al. [32] indicated that the relationships between income inequality and subjective well-being are not influenced by the measure used to assess subjective well-being, but the level of development of the country studied significantly moderates this relationship. Interestingly, in the group of developed countries to which the CEEc belonged, the relationship was negative. Most often, the negative relation between income inequalities and happiness is identified [16, 30, 31]; some studies underline that relationships between income inequities and subjective well-being dimension vary on the country’s inequality levels, and stronger relationships are identified when extremely high- or low-income inequalities characterize income distribution. In countries with higher income inequality, one’s life satisfaction is determined more strongly by income. Specifically, individuals living in an equal society require a minor income increase to achieve a one-point increase in life satisfaction [35]. Haller and Hadler [17] and Tavor et al. [36] showed that happiness is lower in countries with unequal income distribution and very low-income inequalities, characterized by extreme inequality values measured by the Gini index, which in both cases are considered unfair [17, 36]. Moreover, the negative association between income inequalities and happiness can also be considered from an income-class perspective among extreme classes. Namely, high-income and low-income class respondents are less happy because of perceived unfairness in income distribution [37]. However, Easterlin et al. [38] revealed that higher-income individuals have higher life satisfaction. For the lowest income groups, the negative links between life satisfaction and income inequalities were identified by [39]. However, because human beings are aware of the varying contributions of individuals that should be rewarded with differing incomes or profits, moderate inequality should define neither a positive nor negative effect of inequality on happiness [36]. However, some researchers indicate that individual perception of the position in income distribution predicts life satisfaction more strongly than absolute income level [40].

It is broadly believed that increasing income inequalities have negative consequences on other, objective, dimensions of well-being, such as health [14, 41–43], education [44], and environment [43, 45]. Decoster, Minten, and Spinnewijn [46] identified the income gradient in health outcomes and mortality in Belgium, revealing higher mortality between bottom income groups [46]. Deaton described a general health–income gradient, indicating that more affluent people are healthier [42]. However, Wilkinson & Pickett [14] revealed that income inequalities negatively affect health, concluding that narrowing the income gap will improve the health of populations. Moreover, the links between selected well-being dimensions are also studied in the economic literature, e.g., environmental degradation plays a channel through which income distribution affects a society’s health [43]. Also, Ali and Audi identified the negative impact of income inequality and environmental degradation on health [47]. Family investment in early education is important in explaining income inequality and intergenerational income mobility. Because poor children cannot enter universities due to budget constraints in early education, subsidizing early education is the most effective policy to release income inequalities regarding Yang and Qiu [48]. To address relationships between income inequalities and education, Coady and Dizioli concluded with policy suggestions that education expansion will reduce inequality [44].

In conclusion, even though the associations between income inequalities and different dimensions of well-being are inconclusive in the literature, the general findings suggest that, in most cases, the reduction of income inequalities helps improve societies’ well-being when subjective and objective dimensions are considered. We thus hypothesize that

*H1*: *the patterns of relationships between income inequality and well-being dimensions differ between groups of countries with higher (CEE North) and lower (CEE Continental) inequality levels*.
*H2*: *The countries in the delimited groups*, *i*.*e*., *CEE North and CEE Continental*, *with higher and lower inequality levels*, *respectively*, *are homogenous in terms of the interconnectedness between income inequality and well-being dimensions*.

Given the classification into CEE North and CEE Continental, H1 posits that inequality’s effects on well-being dimensions may vary based on whether a country is included in the high or low inequality group, reflecting differences in economic structure and social policies. However, H2 posits that there are similarities in the relationships between income inequalities and well-being dimensions within groups, reflecting the high and low-income inequality levels in the groups.

## Data

### Data characteristics

All variables used in research are expressed in natural logarithms. However, individual variables are represented by several economic processes. The data from 2004 to 2020 are obtained from different databases (Table 1).

**Table 1:** Data description.

	Well-being dimension	Variable	Description	Units	Source
Income inequalities		INEQ	Gini coefficient of equivalized disposable income	Index ranging from 0–100	EU-SILC, Eurostat
Objective well-being	Material dimension	MD	Adjusted gross disposable income of households per capita	Purchasing power standard (PPS) per inhabitant	Eurostat
	Health dimension	HD	Life expectancy of birth	years	World Bank
	Education dimension	EDU	Share of population with tertiary education (levels 5–8), 25–64 years	% share of population 25–64 years	Eurostat
	Environment dimension	ENV	CO2 emissions	metric tons per capita	World Bank
Subjective well-being	Subjective evaluation of one’s life as a whole	sWB	The happiness index is used as a measure of satisfaction with life	Index ranging from 0–10	World Database of Happiness

Even though the term well-being is frequently used synonymously with quality of life [49], in the present study to underline the different dimensions of well-being, we distinguish subjective and following objective aspects, i.e., income, health, education, and natural environment. Subjective well-being, understood as a subjective appreciation of one’s life, is presented by the happiness index.

### Trends in income inequalities in CEEc

CEEc have shared a common trend of increasing income inequalities from the beginning of socio-economic transformation since the demise of communist rule in the late 1980s. However, income inequality mainly increased in the early years of systemic transformation to levels similar to those in many Western European countries [50]. The average Gini index in CEEc was equal to 28.7 in 1993, increased to 30.7 in 2005, and decreased to 28.0 in 2020.

**Fig 1:**
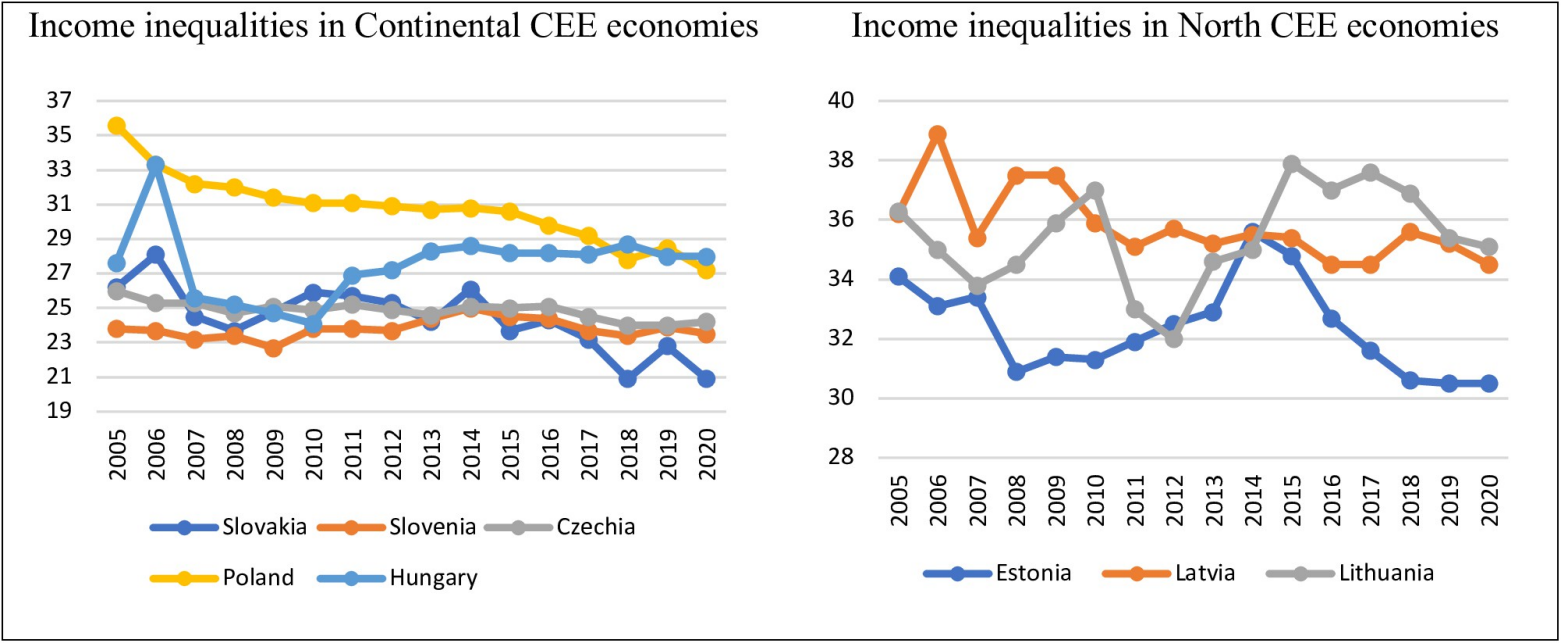
Income inequality trends in CEE Continental and CEE North in 2005–2020 (Gini indices).

The diversity in income inequality in CEEc was either observed at the beginning of the 1990s, and after accession to the EU. Additionally, in 1993, Baltic countries shared much higher income inequalities than, e.g., Poland, Slovak Republic, Czech Republic, and Hungary. In Estonia, Latvia, and Lithuania, the Gini index was, on average, 33.4 in 1993, with the highest level in Estonia and lowest in Latvia, 39.5 and 27.0, respectively [50]. An average level of income inequalities characterized the other CEE was equal to 25.9, with the lowest Gini index in the Slovak Republic (19.5) and the highest Gini index in Slovenia (29.2) in 1993 [50] From the 1990s Hungary, Latvia and Lithuania registered significant increases in inequality. Czech Republic, Slovakia, and Poland maintained stable levels of income inequalities. In Slovakia, income distribution became more equal [51]. After the accession of CEEc to the EU, similar characteristics were observed in CEEc, with the highest income inequalities in the Baltic States and Poland (between 34.1 in Estonia and 38.9 in Latvia) and the lowest in Slovenia (23.8). In 2020, about thirty years from the beginning of the systemic transformation in CEEc, income inequalities decreased in all CEEc except Hungary. The most significant decline was observed in Poland (from 35.6 in 2005 to 27.2 in 2020) [3]–see Fig 1.

Additionally, the diversity in income inequality decreased in CEEc after the beginning of systemic transformation. In 1993, the highest Gini index was more than two times more than the lowest (the Gini index ranged between 19.5 and 39.5 in CEEc). One year after accession to the EU, in the 2005 Gini index ranged between 23.8 and 38.9. Similar diversity continued in the membership in the EU period, but still relatively high was observed in 2020 when the highest Gini index (35.1) was about 1.7 times more than the lowest (20.9).

### Classification of CEE economies regarding the level of income inequalities

According to the observed trends in income inequalities in CEEc and about economic literature, CEE economies were divided into two groups: high and low levels of income inequality. The clustering was conducted based on the average GINI index of the eight CEE economies in 2020 and corresponded to the classification provided by the World Bank [52], Eurofound [4], and Whelan and Maitre [5], who based the classification on Esping-Andersen (1990) [1, 5, 6]. The higher level of income inequalities (higher than the CEEc average) corresponds to the group of economies named CEE North (post-socialist liberal), and the lower level of income inequalities (lower than CEEc average) corresponds to a group of economies–CEE Continental (post-socialist corporatist; see Table 2 and Fig 1).

**Table 2:** Classification of CEE economies.

Group classification*	Group classification **	CEE economies	The level of income inequalities relative to the average of CEE economies***
CEE North	Post-socialist liberal	Estonia, Latvia, Lithuania	Higher
CEE Continental	Post-socialist corporatist	Slovenia, Slovakia, Hungary, Czech Republic, Poland	Lower

* The group classification is based on the [52].

** The group classification is based on [5].

** The average Gini index of 8 CEE economies equals 28.0 [3].

## Methods and empirical results

### VAR model, unit root testing, and residual diagnostics

To investigate the relationship between income inequalities and different dimensions of well-being, the vector autoregression model of order p, VAR(p) is considered [53]:

$$Y_{t}=C+A_{1}Y_{t-1}+A_{2}Y_{t-2}+\cdots +A_{p}Y_{t-p}+\varepsilon_{t}$$

Where Yt is the (n × 1) vector of endogenous variables, C is a constant, Ai–(n × n) is the matrix of the autoregressive coefficients for i = 1, 2, …, p, and εt is an (n × 1) vector of error terms with a zero mean and the variance–covariance matrix Ω.

In this paper, a six-variable VAR model for each economy under study is used. The vector of endogenous variables includes Yt′ = [INEQ, MD, HD, EDU, ENV, sWB], and the description of variables is in Table 1.

Using the VAR model, it is possible to examine the dynamic interactions among all endogenous variables capturing the complex dynamic linkages in economies and to estimate the impact and effects of shocks in each variable on both that variable and other variables. In the context of our study, this means that shocks in the subjective dimension of well-being (happiness index) may affect income inequalities directly or indirectly through other variables such as the material, health, education, or environmental dimension.

**Scheme 1:**
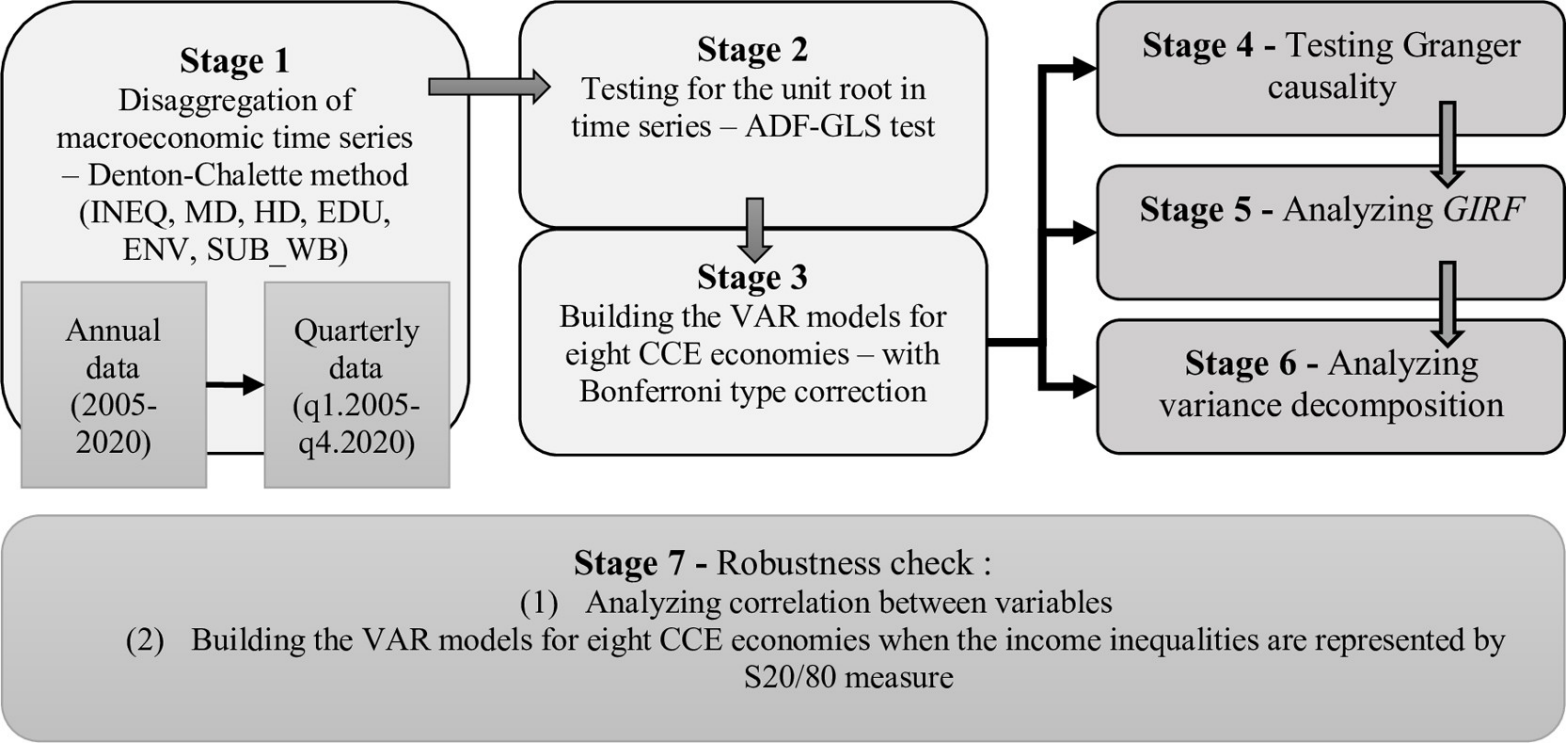
The concept of studying the relationships between income inequalities and different dimensions of well-being.

The relationships between income inequality, and objective and subjective well-being are examined according to Scheme 1. We interpolated annual data to quarterly frequency by employing the Denton-Cholette method [54] in the Eviews software. The sample period is from 2005:Q1 to 2020:Q4. All variables are employed with their natural logarithms form to reduce heteroscedasticity.

All variables are expressed in natural logarithms and are tested for the unit root using the ADF-GLS test. The results indicate that all variables (for all analyzed economies) are integrated in first order (see S2 Table). In five cases (health dimension (HD) for Poland, Slovakia, Czech Republic, education dimension (EDU) for Poland, and income inequalities (INEQ) for Latvia), the ADF-GLS test was insufficient to identify the presence of a unit root, in which case an additional unit root test was used. The Phillips-Perron test shows that at a 10% significance level, a unit root exists in a time series (see S1 Table).

After transforming variables into first differences, the order of the VAR model is chosen using the lag length test. The dummy variables are also included in the VAR model to account for the possible impact of structural changes in time series (like the financial crisis in 2008).

In all cases, the VAR models satisfy the stability condition, which means no root lies outside the unit circle. Generally, we found no evidence of a severe violation of the VAR model, except for a lack of normality due to excess kurtosis, which is not as serious for estimation results as a lack of symmetry in residual distribution [53]. However, the latter is not violated in our VAR models. Furthermore, the VAR residual heteroscedasticity test results show no evidence of variance heteroscedasticity in the models (see S2 Table).

The constructed VAR models were used to perform causality testing in the Granger sense, where multiple performing of chi-squared test is required. The simultaneous testing of multiple hypotheses carries the inherent risk of an elevated significance (α) level, which represents an important challenge in the domain of multiple comparisons. An increase in significance level indicates that the null hypothesis is rejected with greater frequency than is warranted, when it is true. This results in an over-identification of differences, where no differences exist. To mitigate the potential for an increase in α-level, a viable approach is to either adjust the α level or modify the empirical probability (p-value) of the tests in a manner that compensates for the heightened risk of Type I errors. Among the most prominent corrections are the Bonferroni correction and the Sidak correction (1967). In this study, the Sidak correction was employed, which is somewhat more lenient and enables the test’s power to be enhanced. Sidak correction takes the following form [55]:

$$p_{\left(Sidak,i\right)}=1-{\left(1-p_{i}\right)}^{c}$$

where: *p*_*i*_−empirical probability for test statistic, *c*–is number of made comparison.

### Granger causality testing

**Fig 2:**
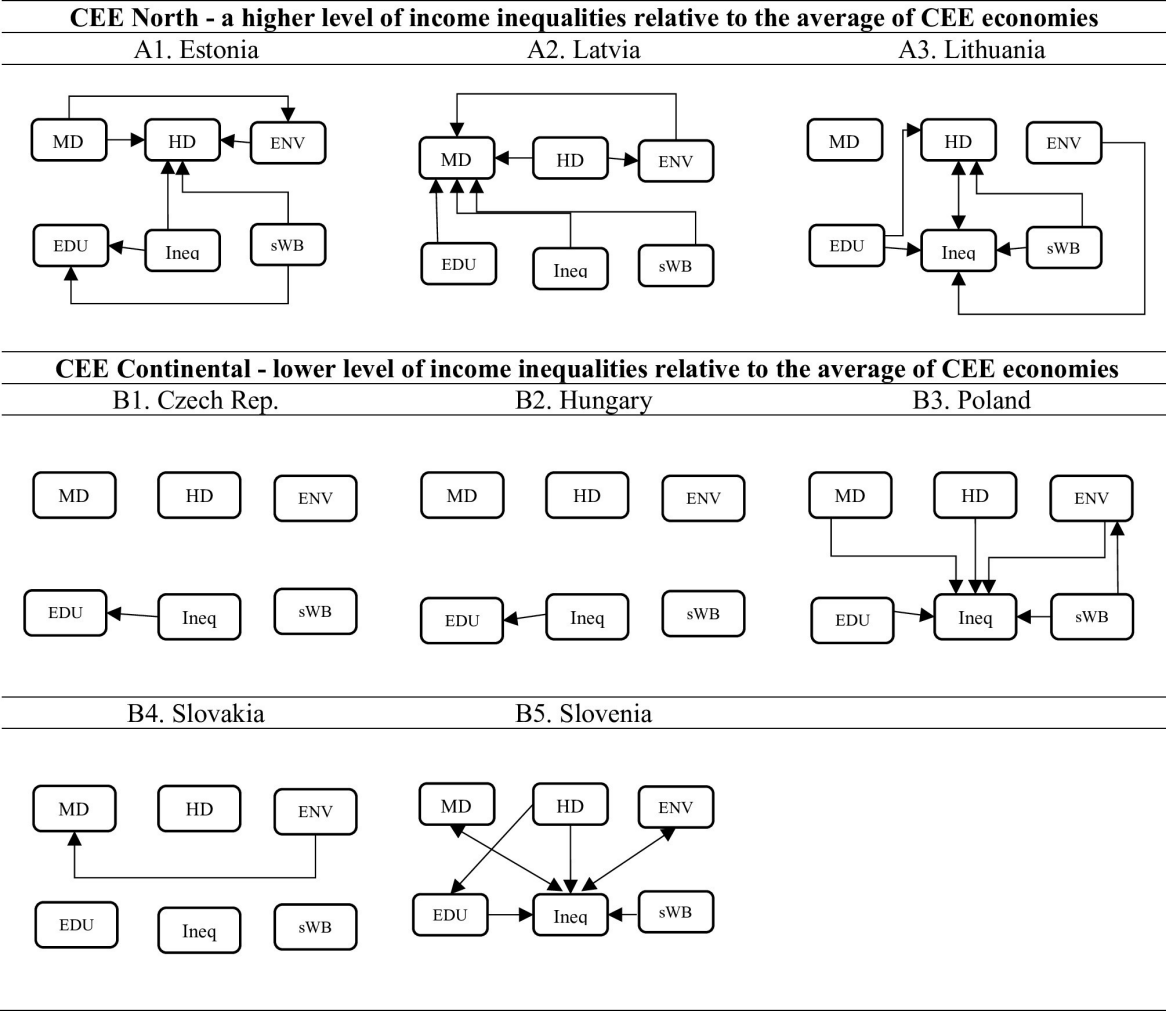
Granger causality maps for eight CEE economies–inequality represented by the Gini index. Source: own presentation based on S3 Table.

Based on the results reported in S3 Table, the Granger causality schemes (see Fig 2) were developed for each analyzed economy. The Granger causality scheme shows the patterns of relationships between income inequalities and well-being dimension (objective and subjective). Also, it allows us to identify the direct and indirect linkages for a system of VAR model equations.

The chi-squared $\left(\chi_{\alpha =5\%}^{2}\right)$ causality test results reveal substantial similarities and differences in connectedness path across the analyzed economies. The results of Granger causality in a group of CEE North economies (consisting of Estonia, Latvia, and Lithuania–Table 2) display numerous significant causal relationships among income inequalities and dimensions of well-being.

In Lithuania, the dimensions of income inequality depend on the environmental, educational, and health dimensions, as well as on subjective perceptions of happiness. At the same time, we observe a significant dependence of the health dimension on the subjective level of happiness, the education dimension, and income redistribution differences (S3 Fig). Regarding the interdependence of the health dimension with the other dimensions analyzed, we find that the Estonian economy is similar to the Lithuanian economy (S2 Fig). On the other hand, the third economy included in the CEE North group of countries–Latvia, shows significant differences in the pattern of connections compared to Lithuania and Estonia, as it is the material dimension of well-being dependent on the other dimensions (i.e., education, environment, health, happiness and income inequality) (S1 Fig). For CEE North economies, there are disparities in the distribution of happiness or life satisfaction, which can result in social and economic inequalities. Only in the case of the Estonian economy can changes in subjective well-being be caused by changes in income inequality. It is important to note that the relationships under examination are structural in nature, exhibiting stable, long-term, and gradual changes at the process level.

Indirect links are also relevant to the links between inequality and dimensions of well-being. In the case of Latvia, no indirect linkages have been identified, while for the economies of Estonia and Lithuania, such linkages have been identified. In Lithuania, the subjective and educational dimensions of well-being are transmitted through the health dimension to income inequality (i.e., *sWB*→*HD*→*INEQ*; *EDU*→*HD*→*INEQ*). On the other hand, in Latvia, the environmental and material dimensions are the most important as they are the ones that link inequality to the health, educational, and subjective dimensions of well-being (i.e. *MD*→*ENV*→*HD*)–see Fig 2A1–2A3.

The group of CEE Continental economies is represented by the Czech Republic, Hungary, Poland, Slovakia, and Slovenia, where the level of income inequalities is lower than the average of CEE economies. Regarding the CEE continental group of countries, a clear division into two sub-groups is evident. In the case of Slovenian and Polish economies substantial causal relationships were identified between well-being dimensions and income inequalities. Poland and Slovenia constitute one group, where income inequalities are dependent on material dimension (MD), health dimension (HD), environmental dimension (ENV), educational dimension (EDU) and happiness index as a subjective well-being dimension (sWB). Significant relation means that changes in objective (MD, HD, EDU, ENV) and subjective dimensions of well-being (happiness index) can predict changes in income inequalities, as well as being a source of reduction or increase of income inequalities in the economy. In the case of Slovenian economy, bi-directional relationships between the income inequalities and material dimension (INEQ↔MD) and environmental dimension (INEQ↔ENV), indicating co-movements in a set of variables. However, in Poland’s subjective well-being (sWB) exerts an indirect influence on income inequality (INEQ) through the environmental dimension (ENV), which is the only identified indirect relation–see Fig 2B3.

In case of the Slovak, Hungarian and Czech’s economies, the analyzed variables are independent of each other, with only few unidirectional relationships indicated. For Hungary and the Czech Republic, there is causal relationship running from income inequality to the education dimension, while in Slovakia the environmental dimension affects the material dimension. This indicates the CEE Continental economies are differentiated, and only Slovenia and Poland show several causal relationships, while the other economies are independent in terms of the causal relationships examined–see Fig 2B1–2B5.

The empirical results of Granger causality indicate that the patterns of interconnectedness in groups of CEE North and CEE Continental countries are not homogeneous. Clusters of groups demonstrate similarities in terms of interdependence between well-being dimensions and income inequality. The CEE North countries form a heterogeneous group with disparate patterns of interdependence. Within the CEE Continental group, Slovenia and Poland represent the initial cluster, exhibiting a notable linkage between income inequality and well-being dimensions. The remaining countries constitute the second cluster, exhibiting a neutrality in relationship between income inequality and the well-being dimensions.

Overall, the results are important because they can help us better understand the relationship between income inequalities and various dimensions of well-being in CEE economies and potentially inform policies to reduce income disparities and promote equitable outcomes in these dimensions.

### Impulse response function

The generalized impulse response function (GIRF) is applied for each economy based on the estimated VAR model. GIRF, conditional on shock (ε_*t*_) and history (ω_*t*-1_) takes the following form [56]:

$$GIRF\left(h,\varepsilon_{t},\omega_{t-1}\right)=E\left[y_{t+h}|\varepsilon_{t},\omega_{t-1}\right]-E\left[y_{t+h}|\omega_{t-1}\right]$$

The GIRF function is the difference between two conditional expectations of *y*_*t+h*_ with a single exogenous shock, and *h* is the forecast horizon. The GIRF analysis [56] allows one to trace out the dynamic response of one variable to one standard deviation shock in the equation of another variable.

To complement the causality analysis, we apply impulse response functions to trace the dynamic response of income inequalities (INEQ) to shocks in well-being and the dynamic response of the dimension of well-being to shocks in income inequalities. S2 Fig shows the results of generalized impulse response functions (GIRF) of changes in INEQ due to one standard error shock in the equations of dimensions of well-being. Also, this figure shows the effect of income inequalities on changes in dimensions of well-being. Although income inequalities respond somewhat differently in each economy, some similar reaction patterns are observed.

#### CEE–North economies

In the case of the Lithuanian economy, the response of income inequalities (INEQ) to a one standard deviation shock in the happiness index (sWB) is negative. This implies that an increase in subjective well-being results in a decrease in income inequalities. However, over the subsequent five-year period, there are some adjustments in INEQ in response to changes in sWB. The initial significant reaction is observed after two years. A comparable response can be observed with regard to changes in the environmental dimension (ENV). The impulse response function indicates that a significant reaction in INEQ will be observable after 1.5 to 3.5 years in the event of significant changes in sWB or ENV. The bidirectional Granger causality between income inequalities and health dimension was confirmed (S3 Table); however, the reaction between both processes is rather symmetrical, with a more significant reaction observed in the case of INEQ (see S1 Fig).

The results of the Granger causality analysis indicate that income inequality is a significant factor in determining objective dimensions of well-being, including health, education, and material dimension in CEE North economies. In the case of the Estonian and Latvian economies, the INEQ has been found to have a more independent character based on empirical research. Furthermore, the observed inequalities have the potential to shape the dimensions of education or health (in Estonia) and the material dimension (in Latvia). The impulse response functions indicate that the reaction of EDU, HD (in Estonia) and MD (in Latvia) is relatively insignificant during the initial two-year period. However, some adjustments can be observed after two to four years following a significant change in INEQ (S1 Fig).

The constructed models for CEE North economies are technically correct, as the random component has a normal distribution and the variance is homoscedastic. The spurious relationship was removed and tests were corrected to account for multiple comparisons. Additionally, the impulse responses indicate the stability of the models, although the direction of the response in some cases is atypical.

#### CEE ‐ Continental economies

In the case of Slovenia and Poland, some similarities in reaction of income inequalities to changes in well-being dimensions are observed. First, the reaction of INEQ to changes in environmental dimension and subjective well-being has significant delay almost 3 years in Slovenia and almost 4 years in Poland. In both countries, the environmental dimension (ENV) and the happiness index (SWB) have the potential to reduce income inequalities (S2 Fig). However, other CEE continental economies (Hungary, Slovakia and the Czech Republic) appear to be more resilient to that factors. In this respect, Slovenia and Poland are more similar to the Lithuanian economy.

The environmental dimension pays a significant role because the policies of European countries targeting CO2 emission reduction may result in increased production costs and consumer prices, as industries and energy sectors are required to adapt to CO2 restrictions through investments in clean-burning fossil fuel technologies or the development of renewable energy. Consequently, these higher costs may impact lower-income consumers disproportionately, as they tend to be more sensitive to price increases.

The reaction of income inequalities in Poland and Slovenia to the increasing change in the dimensions of health (life expectancy ‐ HD) and material well-being (adjusted gross disposable income of households per capita ‐ MD) is mostly negative, which means that these dimensions have the potential to reduce the uneven distribution of income in these economies. Nevertheless, some adjustments may occur with a delay of at least two (in the case of MD) or three (in the case of HD) years. In Poland, the health dimension plays a more substantial role in the reduction of INEQ, as the response is more prominent during the course of the function. This suggests that improving health conditions, including access to specialized medical care, may be an important factor in reducing income inequality. The impulse response function of INEQ to changes in MD and HD indicates a relatively slow and minor reaction for the Slovenian economy. However, it is still capable of reducing inequalities through the health and material dimensions (Fig 2A).

Prominent role in the reduction of income inequalities in Poland and Slovenia also pays an educational dimension, where the reaction of INEQ to changes in EDU are significant and major, while first reactions are evident with almost three or four years of delay. Due to the length of the education process, this lag may be even longer, but the reductive nature of the education dimension is particularly evident in the case of Slovenia and Poland. Income inequality in the other economies seems to be neutral with respect to this dimension.

In the case of the Slovakian, Hungarian, and Czech economies, the income inequalities have been found to have a more independent character based on empirical research, which is similar to two CEE North economies (Latvia and Estonia). Furthermore, the observed inequalities have the potential to shape only the education dimension (in Czech Rep and Hungary), while in Slovakia, the INEQ has a neutral character towards changes in subjective and objective dimensions of well-being.

It is noteworthy that no discernible response was observed in sWB in response to alterations in INEQ. This indicates that the happiness index is not contingent on income inequalities. Consequently, disparate socio-economic processes may influence this subjective well-being dimension.

## Forecast error variance decomposition

Forecast error variance decomposition (FEVD) shows how much of the variation of income inequalities and well-being dimensions are caused by the variations of included variables in the model. In the case of CEE North economies, the FEVD results are presented in A3.1-A3.3 of S3 Fig. The changes in subjective well-being (sWB) account for about 20% of variations in income inequalities in Lithuania. Other 40% of variations in INEQ are explained by objective dimensions of well-being–health, environment and education dimensions (see A3.1, A3-3 in S3 Fig). In Latvia, 15% of material dimension variance is explained by INEQ (A3.2 in S3 Fig). On the other hand, income inequalities are an important factor for explaining variation in education and health dimensions, where INEQ changes account for approximately 70% and 30% of the variance in EDU and HD, respectively (A3.1, A3.3 in S3 Fig). The FEVD results are consistent with causality test results (S2 Table) and impulse response analysis (S1 Fig).

In the case of Poland, the variation in income inequality is explained by changes in the health dimension to a significant extent, with a range of 40–60%. This confirms the significant impact of this dimension on income inequality. The other objective dimensions of well-being account for approximately 20–25% of the variance in the INEQ. In contrast, the subjective dimension of well-being accounts for approximately 15% of the variation in inequality. In the case of Slovenia, the explanations for the observed variation in income inequality are similar to those identified in the Polish case. The health dimension was found to be the most significant contributor, accounting for approximately 40% of the total variation, while the subjective well-being dimension accounted for nearly 30% of the observed inequality. This indicates a similarity in the underlying economic structures of the two countries (S3 Fig).

In regard to the lack of substantial causal relationships between the well-being dimensions and income inequality in the Hungarian, Czech, and Slovak economies, the FEVD results are not interpreted. It can be indicated, however, that in the case of the Hungarian and Czech economies, the contribution of income inequality to explaining the education dimension is approximately 10% and 5%, respectively. This indicates a relatively minor contribution and low significance in the identified causal relationship in Hungary and Czech.

The FEVD results and the Granger causality results (Fig 1 and S2 Table) indicate the important role of well-being dimensions and their impact on income inequalities in the analyzed economies.

### Robustness check

The robustness of the results on the relationship between income inequalities (measured by the Gini index) and dimensions of well-being is tested by applying a correlation coefficient analysis. Additionally, we built the VAR models for each economy where the s20/80 indicator describes the income inequalities. The correlation coefficients are normalized in the interval [–1, 1] and measure the strength of the linear relationships between analyzed variables. Table 3 shows the correlation among macroeconomic variables for eight considered economies.

**Table 3:** Correlation between income inequalities and well-being dimensions in CEE North and CEE Continental.

Variable	CEE North			CEE Continental				
	Estonia	Latvia	Lithuania	Czech Rep.	Hungary	Poland	Slovenia	Slovakia
MD	-0.443	0.255	0.376	-0.198	0.545	-0.065	0.207	-0.238
HD	-0.,239	0.125	-0.037	0.021	0.474	0.474	0.502	0.658
ENV	0.000	-0.060	0.090	-0.244	0.063	-0.525	0.234	-0.334
EDU	0.102	-0.023	0.175	0.028	0.271	-0.118	-0.409	0.238
sWB	0.146	-0.287	-0.294	0.498	0.081	0.120	0.144	0.595

In the case of CEE North economies, it is worth pointing out that the correlation between income inequalities and different dimensions of well-being is rather weak (in some cases moderate) and not always significant. In the Estonian economy, the strongest correlation exists between the material dimension of well-being and income inequalities, where this coefficient takes a value of -0.443 and shows a negative and moderate relation. An increase in income in the dimension of material well-being means a decrease in inequalities (Table 3). Interestingly, in the case of Lithuania and Latvia, the subjective dimension of well-being (represented by the happiness index) is negatively correlated with income inequality at a weak/moderate level. That means the increase in perceived happiness contributes to a decrease in inequality. At the same time, the subjective well-being dimension is a Granger cause for income inequality based on the respective VAR models. The results obtained in the robustness analysis support the results of the main study, where the subjective dimension of well-being plays a pivotal role in the description of income inequalities in Lithuania and Latvia.

In the case of CEE Continental economies, the correlation between income inequalities and objective and subjective well-being is significantly differentiated. The highest correlation exists between the health dimension of well-being and income inequalities in Hungarian, Polish, Slovenian, and Slovakian economies (Table 3). Also, in the case of the Czech Republic and Slovakia, a very important role plays in the happiness index (in the subjective well-being dimension). In all CEE Continental economies, the environmental dimension is important, where the highest correlation takes the value -0,525 (in Poland) because the environmental dimension is strongly linked to the main sectors of the economy, i.e., industry, transportation, and the energy system. An increase in CO_2_ emissions into the atmosphere is associated with a reduction in income inequality (negative correlation in results), as greater production and growth of the economy is associated with increased emissions. However, it is important to reverse this relationship, where it will be possible to reduce income inequality while reducing the emissions of pollutants through appropriate policies and following the principles of sustainable development.

Importantly, using the s20/80 index as a variable describing income inequality in VAR models makes it possible to confirm a number of causal relationships between dimensions of well-being and income inequality. The full results of the causality study and the causality diagrams are available from the article’s authors upon request in ZENODO Repository. Posting these results notably inflates the number of pages of the article, which is already very extensive. The aforementioned relationship also confirms the earlier survey results, indicating that the obtained results are robust.

## Discussion and conclusion

Despite similarities in the CEEc systemic transformation, based on our study, we identified that diversity in the objective income inequalities in those post-socialist states doesn’t play an important role when links between income inequalities and dimensions of well-being are explored. The analysis that was conducted allowed us to answer all the research questions regarding two groups of countries and address the research hypotheses set.

Firstly, the results of our analysis revealed that the interconnectedness between income inequalities and different dimensions of well-being differ between the groups (RQ1). There is significant variation between the CEE North (high-inequality) and CEE Continental (low-inequality) groups regarding the relationship between income inequality and well-being dimensions. In CEE North countries, income inequality more frequently impacts objective well-being aspects, such as health and education, than in CEE Continental countries, aligning with H1 regarding the differences in relationship patterns based on inequality levels.

When it comes to exploring similar patterns inside the groups, CEE North, Estonia, and Lithuania reveal some similarities in the links between health and other well-being dimensions. Within the CEE Continental group, Poland and Slovenia show unique patterns where income inequality is significantly connected to multiple well-being dimensions, especially health and environmental aspects, as well as subjective well-being. However, even though some clusters in terms of similar patterns of the relationships between income inequalities and well-being dimensions can be identified, we concluded that the groups of CEE North and Continental economies are not homogenous regarding how income inequalities are related to well-being dimensions (RQ2). This partly supports H2, indicating similarities within the CEE Continental group but with distinct, intensified interactions in some economies.

Regarding RQ3, subjective well-being (happiness) and health are consistent predictors of income inequality variation in both CEE North and CEE Continental countries. In high-inequality CEE North, subjective well-being has a pronounced impact on income inequality, while in CEE Continental, health plays a larger role.

This means that the income inequality level in the delimited groups of CEEc North and Continental is unimportant for the analyzed relationships. Even though clustering of CEEc is applied in the sense of welfare state typology [1, 5, 6], it does not work for the studied links.

Moreover, regarding Haller and Hadler [17] and Tavor, Gonen, and Spiegel [36], who identified lower happiness in economies with extremely low and high-income inequalities, our results addressed those conclusions in two ways. Firstly, we analyzed the two groups of CEEc divided regarding the level of income inequalities, which, in comparison to the other EU countries, represent the two extremes of income inequalities in the EU and are characterized by a relatively lower level of well-being in comparison to liberal and conservative-corporatist western European countries [7]. Secondly, we analyzed the causality of the links between income inequalities and happiness as one of the dimensions of well-being, revealing different patterns of those links in the delimited groups. Our results regarding income and happiness links were similar to Easterlin [57] and Quispe-Torreblanca et al. [35], revealing that happiness was more often significantly related to income (material dimension of well-being in more unequal societies (CEE North).

Given that concerns over income inequalities in post-socialist states have strongly increased since the demise of the communist system [58] and that income inequalities may negatively affect well-being [14], the research on the links between income inequalities and well-being is particularly important in CEEc. However, given the results of our study revealing that the CEEc classified based on objective income inequality levels are not homogenous when it comes to the links between income inequalities and the dimensions of well-being, the further study we are going to conduct is to consider not only objective but also subjective income inequalities. In particular, we aim to check how the attitudes toward income inequalities are interconnected with well-being dimensions.

The Authors are aware of the limitations of the study, which e.g. are related to the definition adopted and the selection of the well-being dimensions. However, when its links with income inequalities are studied, the two measures of income inequalities, namely the Gini index and income quintile share ratio s80/s20, make the results of our research robust.

## Supporting information

S1 Table Unit root test results (ADF-GLS test). (DOCX)

S2 Table VAR model’s residual diagnostic tests–a multidimensional approach. (DOCX)

S3 Table VAR Granger causality test results. (DOCX)

S1 Fig Reaction between income inequalities and different dimensions of well-being–the results of GIRF for CEE North economies. (DOCX)

S2 Fig Reaction between income inequalities and different dimensions of well-being–the results of GIRF for CEE Continental economies. (DOCX)

S3 Fig Variance decomposition of variables in CEE North and Continental economies. (DOCX)
