# Correction: Multidimensional well-being and income inequality in Central and Eastern Europe: A comparative analysis of CEE North and CEE Continental countries

**DOI:** 10.1371/journal.pone.0329890

**Published:** 2025-08-05

**Authors:** Andrzej Geise, Małgorzata Szczepaniak

There are errors in the Author Contributions. The correct contributions are:

**Conceptualization:** Małgorzata Szczepaniak.

**Methodology:** Andrzej Geise.

**Visualization:** Andrzej Geise.

**Writing – original draft:** Małgorzata Szczepaniak, Andrzej Geise.

**Writing – review & editing:** Małgorzata Szczepaniak, Andrzej Geise.
