# Supplementary material for: Multidimensional well-being and income inequality in Central and Eastern Europe: A comparative analysis of CEE North and CEE Continental countries
Source: PLoS One. 2025 Jan 14;20(1):e0316325. doi: 10.1371/journal.pone.0316325 (PMC11731869; doi:10.1371/journal.pone.0316325)
Supplement: S1 Table — (DOCX) [file pone.0316325.s001.docx]

**S1 Table A1. Unit root test results (ADF-GLS test)**

| Variable | **Czech Rep** | | **Hungary** | | **Estonia** | | **Latvia** | |
| --- | --- | --- | --- | --- | --- | --- | --- | --- |
|  | ***lvl*** | ***diff*** | ***lvl*** | ***diff*** | ***lvl*** | ***diff*** | ***lvl*** | ***diff*** |
| *INEQ* | -1,58 (c+t) | -2,948 (c) *** | -1,291 (c) | -2,127 (c) ** | -1,929 (c+t) | -2,611 (c) *** | -1,952 (c+t) | -0,448 (c) ^m4^ |
| *MD* | -1,994 (c+t) | -2,631 (c) *** | -1,596 (c+t) | -2,659 (c) *** | -1,575 (c+t) | -3,439 (c) *** | -2,158 (c+t) | -3,38 (c) *** |
| *HD* | -0,919 (c+t) | -1,322 (c+t) ^m5^ | -1,341 (c+t) | -1,242 (c) | -0,957 (c+t) | -1,79 (c) * | -0,826 (c+t) | -2,573 (c) ** |
| *EDU* | -1,684 (c+t) | -1,72 (c) * | -1,123 (c+t) | -2,456 (c) ** | -1,974 (c+t) | -2,194 (c) ** | -0,793 (c+t) | -2,158 (c) ** |
| *ENV* | -1,828 (c+t) | -2,559 (c) ** | -1,707 (c+t) | -2,586 (c) ** | -0,715 (c+t) | -3,913 (c) *** | -1,457 (c+t) | -3,681 (c) *** |
| *SUB_WB* | -0,751 (c+t) | -1,63 (c) * | -0,904 (c+t) | -3,195 (c) *** | -2,046 (c+t) | -3,556 (c) *** | -1,843 (c+t) | -4,399 (c) *** |
| 1% | -3,759 | -2,609 | -3,759 | -2,609 | -3,759 | -2,609 | -3,759 | -2,609 |
| 5% | -3,180 | -1,947 | -3,180 | -1,947 | -3,180 | -1,947 | -3,180 | -1,947 |
| 10% | -2,881 | -1,613 | -2,881 | -1,613 | -2,881 | -1,613 | -2,881 | -1,613 |
| Variable | **Lithuania** | | **Poland** | | **Slovakia** | | **Slovenia** | |
|  | ***lvl*** | ***diff*** | ***lvl*** | ***diff*** | ***lvl*** | ***diff*** | ***lvl*** | ***diff*** |
| *INEQ* | -1,571 (c+t) | -3,151 (c) *** | -1,827 (c+t) | -2,049 (c) ** | -1,14 (c+t) | -2,275 (c) ** | -1,140 (c+t) | -2,275 (c) ** |
| *MD* | -2,167 (c+t) | -4,233 (c) *** | -0,674 (c+t) | -2,526 (c) ** | -0,895 (c+t) | -2,722 (c) *** | -2,161 (c+t) | -2,411 (c) ** |
| *HD* | -2,084 (c+t) | -3,851 (c) *** | -1,068 (c+t) | -0,649 (c) ^m1^ | -0,635 (c+t) | -1,249 (c) ^m3^ | -0,429 (c+t) | -1,847 (c) * |
| *EDU* | -0,894 (c+t) | -1,671 (c) * | -0,437 (c+t) | -1,266 (c) ^m2^ | -2,48 (c+t) | -2,45 (c) ** | -2,262 (c+t) | -1,809 (c) * |
| *ENV* | -1,725 (c+t) | -3,085 (c) *** | -2,217 (c+t) | -3,566 (c) *** | -1,461 (c+t) | -2,823 (c) *** | -1,797 (c+t) | -2,704 (c) *** |
| *SUB_WB* | -1,822 (c+t) | -3,437 (c) *** | -1,992 (c+t) | -2,455 (c) ** | -1,51 (c+t) | -3,036 (c) *** | -1,400 (c+t) | -2,215 (c) ** |
| 1% | -3,759 | -2,609 | -3,759 | -2,609 | -3,759 | -2,609 | -3,759 | -2,609 |
| 5% | -3,180 | -1,947 | -3,180 | -1,947 | -3,180 | -1,947 | -3,180 | -1,947 |
| 10% | -2,881 | -1,613 | -2,881 | -1,613 | -2,881 | -1,613 | -2,881 | -1,613 |

^m1^ -Poland – HD – Phillips-Perron test statistic = -2.596 (p=0.0992) I(1); ^m2^ -Poland – EDU - Phillips-Perron test statistic = -2.545 (p=0.1) I(1); ^m3^ -Slovakia – HD – Phillips-Perron test statistic = -2.949 (p=0.046) I(1); ^m4^ -Latvia – INEQ – Phillips-Perron test statistic =-3.265 (p=0.021) I(1); ^m5^ -Czech Republic – HD – Phillips-Perron test = -3.221 (p=0.09) I(1);
