# Supplementary material for: Multidimensional well-being and income inequality in Central and Eastern Europe: A comparative analysis of CEE North and CEE Continental countries
Source: PLoS One. 2025 Jan 14;20(1):e0316325. doi: 10.1371/journal.pone.0316325 (PMC11731869; doi:10.1371/journal.pone.0316325)
Supplement: S2 Table — (DOCX) [file pone.0316325.s002.docx]

**S1 Table A2. VAR model's residual diagnostic tests – a multidimensional approach**

| Residuals diagnostic test | **Czech Republic** | **Estonia** | **Hungary** | **Latvia** |
| --- | --- | --- | --- | --- |
| Joint $\chi^{2}$ test for skewness | 2,55 (p=0,863) | 10,84 (p=0,093) | 8,825 (p=0,18) | 5,706 (p=0,457) |
| Joint $\chi^{2}$ test for kurtosis | 25,94 (p=0,0002) *** | 25,99 (p=0,0002) *** | 37,36 (p=0,001) *** | 16,29 (p=0,012)  ** |
| Joint JB test | 28,49 (p=0,0047) *** | 36,84 (p=0,0002) *** | 46,18 (p=0,001) *** | 21,99 (p=0,038)  ** |
| Joint $\chi^{2}$ test | 1048,4 (p=0,183) | 1126,3 (p=0,229) | 1052,9 (p=0,295) | 959,7 (p=0,86) |
| Residuals diagnostic test | **Lithuania** | **Poland** | **Slovenia** | **Slovakia** |
| Joint $\chi^{2}$ test for skewness | 3,122 (p=0,793) | 4,035 (p=0,672) | 9,796 (p=0,134) | 5,673 (p=461) |
| Joint $\chi^{2}$ test for kurtosis | 6,674 (p=0,352) | 18,69 (p=0,005) *** | 56,19 (p=0,01) *** | 16,41 (p=0,012) ** |
| Joint JB test | 9,796 (p=0,634) | 22,73 (p=0,03) ** | 65,98 (p=0,01) *** | 22,08 (p=0,04) ** |
| Joint $\chi^{2}$ test | 1026,4 (p=0,337) | 1038,4 (p=0,247) | 1033,5 (p=0,282) | 1001,5 (p=0,552) |

***, **, - significance at α=0,01; α=0,05; respectively.

Source: own calculations
