# Supplementary material for: Multidimensional well-being and income inequality in Central and Eastern Europe: A comparative analysis of CEE North and CEE Continental countries
Source: PLoS One. 2025 Jan 14;20(1):e0316325. doi: 10.1371/journal.pone.0316325 (PMC11731869; doi:10.1371/journal.pone.0316325)
Supplement: S3 Table — (DOCX) [file pone.0316325.s003.docx]

**S1 Table A3. VAR Granger causality test results**

| Czech Republic | | | | | | |
| --- | --- | --- | --- | --- | --- | --- |
|  | INEQ | MD | HD | EDU | ENV | SUB_WB |
| INEQ | - | 3,076 | 1,366 | 19,59 *** | 1,802 | 2,004 |
| MD | 6,456 | - | 4,935 | 4,629 | 0,429 | 1,347 |
| HD | 0,285 | 1,331 | - | 0,246 | 0,253 | 0,929 |
| EDU | 1,358 | 0,765 | 10,311 | - | 1,590 | 0,441 |
| ENV | 11,474 | 2,189 | 3,803 | 5,859 | - | 2,283 |
| SUB_WB | 8,343 | 2,942 | 2,502 | 14,61 | 4,879 | - |
| Estonia | | | | | | |
| INEQ | - | 1,957 | 17,24 *** | 21,89 *** | 13,20 | 13,002 |
| MD | 1,826 | - | 18,49 *** | 9,798 | 20,29 *** | 1,929 |
| HD | 2,641 | 3,093 | - | 14,72 | 7,531 | 9,919 |
| EDU | 2,732 | 2,309 | 12,001 | - | 3,693 | 6,16 |
| ENV | 0,498 | 2,509 | 29,69 *** | 13,23 | - | 3,074 |
| SUB_WB | 0,859 | 3,311 | 21,66 *** | 25,66 ** | 13,29 | - |
| Hungary | | | | | | |
| INEQ | - | 1,247 | 0,257 | 8,395 ** | 0,412 | 1,083 |
| MD | 1,301 | - | 0,721 | 0,535 | 0,461 | 1,392 |
| HD | 1,507 | 4,054 | - | 4,326 | 1,139 | 2,445 |
| EDU | 2,859 | 6,756 | 1,03 | - | 6,943 | 4,990 |
| ENV | 0,041 | 2,129 | 0,494 | 0,834 | - | 2,996 |
| SUB_WB | 0,487 | 0,895 | 2,115 | 1,633 | 3,013 | - |
| Latvia | | | | | | |
| INEQ | - | 25,15 *** | 4,05 | 0,775 | 5,299 | 4,026 |
| MD | 9,73 | - | 4,027 | 0,812 | 9,91 | 4,192 |
| HD | 1,656 | 63,79 *** | - | 0,685 | 23,31 *** | 3,921 |
| EDU | 14,42 | 18,78 *** | 2,394 | - | 6,501 | 2,821 |
| ENV | 13,63 | 23,14 *** | 2,032 | 0,912 | - | 1,374 |
| SUB_WB | 8,053 | 17,73 *** | 1,608 | 0,249 | 9,239 | - |
| Lithuania | | | | | | |
| INEQ | - | 7,769 | 36,48 *** | 8,652 | 15,37 | 1,733 |
| MD | 10,84 | - | 5,479 | 4,292 | 14,97 | 9,963 |
| HD | 43,03 *** | 7,037 | - | 5,847 | 3,642 | 8,079 |
| EDU | 38,52 *** | 14,51 | 28,53 *** | - | 14,103 | 1,492 |
| ENV | 58,51 *** | 9,019 | 14,47 | 7,901 | - | 12,03 |
| SUB_WB | 70,18 *** | 8,312 | 53,17 *** | 7,824 | 9,703 | - |
| Poland | | | | | | |
| INEQ | - | 5,648 | 1,767 | 1,14 | 3,462 | 13,25 |
| MD | 21,51 *** | - | 4,179 | 6,06 | 0,654 | 3,743 |
| HD | 22,89 *** | 3,571 | - | 1,588 | 1,203 | 7,976 |
| EDU | 16,7 *** | 1,788 | 7,905 | - | 5,552 | 11,67 |
| ENV | 31,52 *** | 5,987 | 3,637 | 1,705 | - | 10,32 |
| SUB_WB | 18,96 *** | 0,783 | 2,633 | 1,361 | 16,13 *** | - |
| Slovenia | | | | | | |
| INEQ | - | 24,06 *** | 5,23 | 13,89 | 31,31 *** | 2,556 |
| MD | 20,28 *** | - | 4,155 | 11,49 | 6,04 | 3,523 |
| HD | 23,24 *** | 3,598 | - | 16,56 *** | 9,297 | 1,139 |
| EDU | 20,2 *** | 1,412 | 2,575 | - | 1,703 | 2,374 |
| ENV | 34,77 *** | 9,609 | 2,304 | 8,609 | - | 1,175 |
| SUB_WB | 36,18 *** | 3,976 | 6,371 | 8,615 | 5,001 | - |
| Slovakia | | | | | | |
| INEQ | - | 6,111 | 6,153 | 2,59 | 2,222 | 4,872 |
| MD | 3,265 | - | 1,782 | 0,719 | 8,578 | 8,494 |
| HD | 3,235 | 7,288 | - | 2,272 | 4,39 | 3,062 |
| EDU | 12,6 | 2,573 | 6,692 | - | 2,412 | 4,477 |
| ENV | 5,261 | 17,03 *** | 1,715 | 1,037 | - | 5,388 |
| SUB_WB | 2,781 | 4,059 | 3,441 | 1,853 | 0,749 | - |
