# Supplementary material for: Multidimensional well-being and income inequality in Central and Eastern Europe: A comparative analysis of CEE North and CEE Continental countries
Source: PLoS One. 2025 Jan 14;20(1):e0316325. doi: 10.1371/journal.pone.0316325 (PMC11731869; doi:10.1371/journal.pone.0316325)
Supplement: S1 Fig — (DOCX) [file pone.0316325.s004.docx]

**S1 Fig. A1. Reaction between income inequalities and different dimensions of well-being – the results of GIRF for CEE North economies**

| **Estonia** | | | | | |
| --- | --- | --- | --- | --- | --- |
| Reaction of income inequalities (INEQ) to changes in different dimensions of well-being |  |  |  |  |  |
| Reaction of different dimensions of well-being to changes in income inequalities (INEQ) |  |  |  |  |  |
| **Latvia** | | | | | |
| Reaction of income inequalities (INEQ) to changes in different dimensions of well-being |  |  |  |  |  |
| Reaction of different dimensions of well-being to changes in income inequalities (INEQ) |  |  |  |  |  |
| **Lithuania** | | | | | |
| Reaction of income inequalities (INEQ) to changes in different dimensions of well-being |  |  |  |  |  |
| The reaction of different dimensions of well-being to changes in income inequalities (INEQ) |  |  |  |  |  |
