# Supplementary material for: Multidimensional well-being and income inequality in Central and Eastern Europe: A comparative analysis of CEE North and CEE Continental countries
Source: PLoS One. 2025 Jan 14;20(1):e0316325. doi: 10.1371/journal.pone.0316325 (PMC11731869; doi:10.1371/journal.pone.0316325)
Supplement: S2 Fig — (DOCX) [file pone.0316325.s005.docx]

**S1** **Fig. A2. Reaction between income inequalities and different dimensions of well-being – the results of GIRF for CEE Continental economies**

| **Czech Republic** | | | | | |
| --- | --- | --- | --- | --- | --- |
| Reaction of income inequalities (INEQ) to changes in different dimensions of well-being |  |  |  |  |  |
| Reaction of different dimensions of well-being to changes in income inequalities (INEQ) |  |  |  |  |  |
| **Hungary** | | | | | |
| Reaction of income inequalities (INEQ) to changes in different dimensions of well-being |  |  |  |  |  |
| Reaction of different dimensions of well-being to changes in income inequalities (INEQ) |  |  |  |  |  |
| **Poland** | | | | | |
| Reaction of income inequalities (INEQ) to changes in different dimensions of well-being |  |  |  |  |  |
| Reaction of different dimensions of well-being to changes in income inequalities (INEQ) |  |  |  |  |  |
| **Slovakia** | | | | | |
| Reaction of income inequalities (INEQ) to changes in different dimensions of well-being |  |  |  |  |  |
| Reaction of different dimensions of well-being to changes in income inequalities (INEQ) |  |  |  |  |  |
| **Slovenia** | | | | | |
| Reaction of income inequalities (INEQ) to changes in different dimensions of well-being |  |  |  |  |  |
| Reaction of different dimensions of well-being to changes in income inequalities (INEQ) |  |  |  |  |  |

INEQ – *income inequalities*, MD – *material dimension*, HD – *health dimension*, EDU – *education dimension*, ENV – *environmental dimension*, SUB-WB – *subjective well-being*
