# Supplementary material for: Multidimensional well-being and income inequality in Central and Eastern Europe: A comparative analysis of CEE North and CEE Continental countries
Source: PLoS One. 2025 Jan 14;20(1):e0316325. doi: 10.1371/journal.pone.0316325 (PMC11731869; doi:10.1371/journal.pone.0316325)
Supplement: S3 Fig — (DOCX) [file pone.0316325.s006.docx]

**S1 Fig. A3. Variance decomposition of variables in CEE North and Continental economies**

| A3.1 CEE North - Estonia   | | A3.2 CEE North - Latvia   |
| --- | --- | --- |
| A3.3 CEE North - Lithuania   | | A3.4 CEE Continental – Czech Republic   |
| A3.5 CEE Continental – Hungary   | A3.6 CEE Continental – Poland   | |
| A3.7 CEE Continental – Slovakia   | A3.8 CEE Continental – Slovenia   | |
